# “Shock tactics”, ethics, and fear. An academic and personal perspective on the case against ECT

**DOI:** 10.1192/bjp.2021.116

**Published:** 2022-03-01

**Authors:** Tania Gergel

**Affiliations:** Institute of Psychiatry, Psychology, and Neuroscience, King’s College London

**Keywords:** Electroconvulsive therapy (ECT), depression, bipolar disorder, lived experience, ethics, involuntary treatment

## Abstract

Despite extensive evidence for its effectiveness, ECT remains the subject of fierce opposition from those contesting its benefits and claiming extreme harms. Alongside some reflections on my experiences of this treatment, I examine the case against ECT, and find that it appears to rest primarily on unsubstantiated claims about major ethical violations, rather than clinical factors such as effectiveness and risk.

## Introduction

A recent review discussing the efficacy and safety of modern electroconvulsive therapy (ECT) finds that it is still the “most effective treatment for severe, psychotic or treatment-resistant depression” (1). While ECT is viewed by many clinicians and recipients as indispensable in treating debilitating and “life-threatening” severe mental illness (2,3), it nevertheless remains, arguably, the most stigmatised, misunderstood, contested, and feared psychiatric or perhaps even medical, treatment. A few days after publication of the review, a short and sensationalist newspaper article, “Shock Tactics” (4), directed anyone “considering having a big electric shock passed through your brain” towards a *Psychology Today* article by an influential academic ECT-opponent, disputing efficacy and calling for urgent review of a treatment with “risks of brain damage and death” (5). As a researcher focusing on medical ethics and law, but also someone with considerable lived experience of receiving ECT, my aim here is to examine the nature and validity of the extreme and often vitriolic opposition to this treatment.

Probably the strongest feeling engendered by the notion of ECT is fear. ECT involves an electrical charge being passed through the brain to induce a seizure and cause a radical shift of mental state. Perhaps unsurprisingly, this description itself might sound alien, scientistic, and frightening. Added to this are multiple cultural and media representations situating ECT firmly within the ‘dark side’ of psychiatry (6,7). Most well-known is the iconic 1975 film, *One Flew Over the Cuckoo’s Nest*, portraying psychiatry as a misused tool of repressive social control. Jack Nicholson’s character, who is not mentally unwell, forcibly receives ECT, without anaesthetic, as punishment for insubordinate behaviour. The effects of this treatment can become easily conflated with the gruelling final scene showing Nicholson’s near-vegetative state, resulting from a psycho-surgical procedure not shown and no longer practised. The takeaway impression of ECT is as a sadistic and illegitimate process, punitive rather than therapeutic, and capable of, effectively, destroying the brain. No famous depictions of ECT within contemporary psychiatric practice exist to counter these images, demonstrating the severity of the conditions it treats and its potential benefits. No wonder that ECT remains an object of fear. Yet, for myself, as for many others for whom ECT has been a life-saving treatment, the greatest fear surrounding ECT is that it might one day be inaccessible or abolished.

Before examining the anti-ECT position, I present some potential “conflict of interests” alongside some credentials for my ability to offer a balanced view about psychiatric practice, ethics, and law. For me, personally, the benefits of ECT have been immeasurable in treating a severe and dangerous mixed-affective presentation of bipolar disorder, which remained, until very recently, steadfastly resistant to any acute or maintenance psychopharmacological treatment or psychotherapeutic intervention. I first received ECT aged 21, after over a year of failed treatments and hospitalisations. Eight bi-weekly ECT treatments were for myself and my family the ‘miracle cure’ allowing me to reengage with life and return to University to complete my degree. Treatment was not frightening, and I experienced no significant side effects. Despite receiving over 150 ECT treatments over the years, I have noticed no deterioration of intellectual ability or capacity to build new memories and have been able successfully to resume my academic career. I was also on the Royal College of Psychiatrist’s ECT Accreditation Service advisory committee for 6 years.

However, my experience and views of ECT and psychiatry are not universally positive. I have sustained considerable autobiographical memory loss from later treatments, causing both psychological and practical difficulties, and would never minimise or deny the views of those for whom side effects have been severe and debilitating. While I myself, when well, condone and accept the need for a treatment I often resist while unwell, amongst my many experiences of ECT were instances where treatment and enforcement were mismanaged. More generally, my own academic work often involves critique of contemporary psychiatry and mental health law.

## The case against ECT

The case for suspension or abolition of ECT is usually argued in terms of three main clinical transgressions: lack of evidence for effectiveness; minimisation or even denial of severe side effects; treatment without informed consent. However, close examination suggests that ethical rather than clinical concerns dominate anti-ECT critiques. A pervasive *One Flew Over the Cuckoo’s Nest-type* image emerges of deliberate concealment and human rights violations.

### Claim 1 – lack of evidence for effectiveness

A 2020 review by prominent ECT critics concludes “There is no evidence that ECT is effective for… its target diagnostic group—severely depressed people, or for suicidal people, people who have unsuccessfully tried other treatments first, involuntary patients, or children and adolescents” (8). Such claims, common within anti-ECT literature (9,10), seem strange and are easily challenged, given considerable evidence and abundant patient and clinical testimonies to major benefits (1,3,11–15), including many calling for ECT’s use not to be restricted to ‘last resort’ treatment (1,3,11,12,15). Research on ECT’s effectiveness is too extensive to summarise or assess here. The critical 2020 review only considered studies between 1956 and 1985, with many of its findings highly disputable, particularly in a modern context (15). These points aside, however, let us consider the broader implications of this anti-ECT viewpoint.

The first question must surely be motivation. Around 1.4 million people worldwide receive ECT annually (1). In psychiatric terms, ECT is relatively costly and complex, in most countries involving general anaesthesia, with estimates of annual treatment costs which “can exceed $10 000” (14). If, after 80 years of ECT, there really was no evidence for effectiveness, why would healthcare providers continue funding ECT and what would psychiatrists stand to gain, especially in the face of such acrimonious criticism?

Moreover, claiming that psychiatry knowingly inflicts an invasive medical treatment with potentially serious side effects and no evidence of substantive therapeutic benefits implies a global breach of core medical ethical principles. Not only would this violate both beneficence and nonmaleficence, but seemingly also justice, through allocating limited resources to expensive and ineffective treatments.

Moreover, deliberately misleading patients about therapeutic benefits would surely negate ‘informed’ consent and autonomous decision-making concerning treatment. While psychiatry may sometimes involve errors of clinical judgement, the idea that so many medical practitioners are complicit in breaching fundamental professional ethics seems implausible and devoid of apparent motivation.

### Claim 2 – minimisation or even denial of severe side effects

ECT opponents claim that psychiatry fails to acknowledge the extent, severity, or even existence of severe potential side effects from ECT, including “brain damage”, “mortality”, and “traumatic impact on the brain” (10). However, as with lack of effectiveness, claims that ECT has such side effects, which are deliberately and collectively concealed, denied, or minimised by psychiatrists, once again implies multiple seemingly implausible and unmotivated ethical violations.

It is widely acknowledged that ECT’s most substantial side effect can be “retrospective autobiographical memory” loss and the substantial research exploring ways to reduce retrograde amnesia indicates, very clearly, that psychiatry is neither ignoring nor denying this issue (1,16,17).

Historically, this phenomenon was underacknowledged or even denied (16) and some clinicians, as I myself have witnessed, may still fail to attribute sufficient weight to its nature and impact. While guidance materials and clinical decision-making now usually include consideration and information about such side effects, a desire to emphasise potential benefits may lead to insufficient attention being devoted to issues surrounding retrograde amnesia. For example, the new Royal College of Psychiatrists ECT information leaflet mentions the possibility of “permanent” gaps under “Short-term” rather than “Long-term” side effects (18). Assessing memory issues is further complicated by the difficulties of differentiating residual cognitive impairment resulting from depression from the effects of ECT, which can itself help to relieve these impairments (19).

I have, myself, experienced such memory loss within two perinatal periods. For various reasons, I have twice needed multiple courses of bi-weekly and bilateral ECT within a period of three to four years. Such extensive treatment is unusual and may make my experience of memory loss greater than usual (16). My lasting memory loss relating to people, events, and periods of my life can be difficult both emotionally and practically. I have found various ways to manage this amnesia and am extremely fortunate to have support from multiple people who understand and help to fill in the gaps. For myself and many others (3), although not for everyone, benefits of treatment have undoubtedly outweighed these costs. Beyond these autobiographical memory gaps, however, no clinical evidence supports common accusations of permanent ‘brain damage’, physical damage, or major fatality risk (1,5,9,10,12,22).

### Claim 3 – excessive use of involuntary treatment

A final major concern is the proportion of patients receiving ECT without providing informed consent, usually described by ECT opponents using language implying physical coercion (8). Informed consent will, of course, always be contentious in relation to psychiatry, given common international use of legally sanctioned involuntary treatment. The United Nations Committee on the Rights of Persons with Disabilities, for example, call for abolition of all involuntary treatment (General Comment 1). Yet, with ECT, such concerns appear to extend beyond straightforward questions about ethical validity of involuntary treatment.

Multiple factors might justify administering ECT using statutory measures to allow treatment without informed consent. ECT is increasingly used for severe, life-threatening depression, and treatment-resistant illness, often including psychotic features, catatonia, or prolonged mania. Given probable severity of symptoms and concomitant likelihood of impaired decision-making abilities or extreme risk, informed consent may well not be possible (23,24). In such cases failure to use statutory provision authorising substitute decision-making would itself be unethical, and safeguards surrounding involuntary use of ECT within mental health legislation are typically more stringent than for other forms of treatment. Although the type of physical coercion suggested by ECT critics is not typically involved, such cases would be classified as ‘involuntary’, and a recent dataset from UK clinics reports that 46.7% of patients were formally detained when starting acute ECT treatment, with 41% lacking decision-making capacity to consent to treatment (25). However, there has been a well-documented and alarming rise in the use of formal detention in England and Wales, with a national report showing that, by 2016/7, 80% of adult psychiatric inpatients and 100% of older inpatients (65+) were formally detained in some areas (26). By comparison figures for ECT might even be seen as relatively low given that detention figures amongst acute ECT patients suggest that over half of ECT recipients received treatment having provided informed consent, even though it is likely that the majority were inpatients (18),. Moreover, evidence suggests that patients often regain capacity to consent during a course of ECT and consent to further treatment (23,25), with many involuntary patients retrospectively assessing treatment as helpful (23,27), an experience which I myself have shared.

## Cost-benefit analysis: which factors are often omitted by the anti-ECT lobby?

Accusations of ethical violations through ineffective treatment, concealed side effects, and excessive involuntary treatment seem unconvincing. Moreover, while treatment decisions involve informed cost-benefit analysis (1,3,16), ECT opponents often deemphasise, omit, or even misrepresent details about the treatment process and conditions treated, despite their frequent accusations of obfuscation and concealment amongst ECT practitioners.

In almost all countries, ECT now involves general anaesthesia and a muscle relaxant to prevent major physical convulsion (1,3). In the UK, for example, ECT staff are trained to answer any questions or concerns, provide calming environments both pre- and post-treatment, and conduct physical and cognitive checks (28). For me, when severely unwell, my fears concerning ECT stem entirely from persecutory delusions about “brain-control”, rather than fear of the physical process itself. Most importantly, perhaps, ECT opponents rarely describe the realities of conditions treated by ECT, Unfortunately, terminology used to defend ECT, such as ‘debilitating’, ‘depression’, or even ‘life-threatening’, barely evokes the experience of severe affective disorders or their potential consequences.

Though hard to articulate, I offer some personal examples to try to demonstrate the lived experience and dangers of such conditions and reasons for prescribing ECT. When becoming severely unwell, I suddenly enter an internal world utterly detached from everything and everyone around me. The US psychiatrist Jamison’s description of her own mixed affective state prior to attempting suicide has always resonated deeply - her mind a “murderous cauldron” her body “uninhabitable”, “raging and weeping and full of destruction and wild energy gone amok” (29). For me, “tortuous energy” is underpinned by manic grandiosity and invincibility, with intermittent euphoria pushing me towards enlightenment, but accompanied by terrifying paranoia. This lethal combination is all the more dangerous, usually veiled under a deceptive presentation of calm lucidity.

During the final trimester of my second pregnancy, I descended rapidly into these familiar patterns. I clearly needed ECT, although I did not want this or any other treatment. Why was this? I was bombarded by thoughts, voices, and signs telling me that my psychiatrist, whom I deeply trust and respect, was masterminding a conspiracy to control my mind and prevent me from fulfilling my destiny, making any treatment compliance an act of cowardly capitulation. Nevertheless, like Jamison (29), I had written advance documentation, requesting ECT, administered involuntarily if necessary, if I became severely unwell. In fact, the perinatal risks associated with bipolar disorder and my reliance on ECT are so great that my decision to try for a second child had been heavily contingent on the availability of ECT in the perinatal period.

The literature on ECT in pregnancy is, understandably, limited, but points towards its safety and effectiveness (11,12,30). I received 12 bi-weekly treatments during the last trimester of pregnancy. Treatment took place in the main theatres, with a midwife and obstetric team present, along with full foetal monitoring and provision for emergency delivery. The ECT team were incredible and took me through the process with great compassion, acknowledging and doing everything possible to help me manage my fear. After the 10^th^ treatment, during the 36^th^ week of pregnancy, there was a sudden and dramatic remission of the severe symptoms and psychosis. Just as rapidly as reality had vanished, it returned. The last treatment was at 38 weeks and I gave birth to a healthy child 2 weeks later, who started school this year.

## Conclusion

On examination, academic opposition to ECT appears generally to rest on unsubstantiated claims of ethical violations, some of which its opponents themselves may even perpetrate. Opposition comprises a small but vocal cohort, mainly subscribing to an ideological agenda rarely mentioned within specifically anti-ECT literature (11,15), rejecting any medical understanding of mental illness and frequently questioning psychiatric motives. The critical 2020 review appears within the official journal of an international society centred on the premise that mental illnesses “should not be considered medical problems and traditional medical treatment is not a solution” (https://psychintegrity.org) (8). Very similar views are espoused, for example, on other sites hosting anti-ECT literature such as ‘Behaviourism and Mental Health’ and the ‘Council for Evidence-based Psychiatry’ (http://cepuk.org/).

Based on prejudicial and unjustified assumptions about the intrinsic illegitimacy and immorality of psychiatry, many anti-ECT academics simply assume a lack of credibility in the evidence and testimonies presented by psychiatrists. Similar assumptions about intrinsic vulnerabilities or credulity lead to dismissal or even discrimination against ECT advocates who, like myself, claim to have benefited from the treatment. As Dukakis writes in a thoughtful collection of testimonies from those who have benefited from ECT, including her own: “I fully expect to be attacked. I feel like I am putting a target on my back for ECT’s many critics” (3). Moreover, the views and utter intransigence of calls for suspension or abolition of ECT take no account of potential harms from depriving those helped by ECT treatment and deterring those who are severely unwell from considering treatment which could help to relieve their suffering.

Public perceptions of ECT may well still be dominated by a *One Flew Over the Cuckoo’s Nest* image. Currently, the sensationalist and flawed views of the academic anti-ECT lobby continue to bolster such damaging and unjustified public perceptions and media discussion, rendering it unlikely that any supporting evidence for ECT will ever receive balanced consideration. No matter how much evidence is presented in journals, unless psychiatry is proactive in educating people about ECT and is helped, rather than hindered, by the media, ECT’s ‘image problem’ will persist. The stigma surrounding ECT means “that its use is severely limited, and its merits are neglected or even denied” (11), with even those psychiatrists who recognise its effectiveness deterred from prescribing ECT and training others (3).

My arguments are in no way intended to deny any historic or even contemporary instances of misuse (11), or to negate the views of service users who have experienced harm from ECT, either without any benefits, or with benefits which cannot outweigh the damage. However, any rights-based approach must surely recognise the rights of individuals to conduct their own cost-benefit analysis and to have available to them a treatment with the potential to alleviate severely debilitating and dangerous symptoms (3,11).

From a personal perspective, ECT does not cure bipolar disorder and the condition is for me, as for so many others, an ongoing challenge. I am incredibly lucky to have levels of social, clinical, and material support unavailable to many. I am aware of the high probability that I may one day become severely unwell again. I am also aware that, if I do, I will need ECT and, when I receive the first treatments, there may well be some element of coercion, whether formal or informal. Almost certainly, I will experience some degree of memory loss. But today I am alive. I have two happy and healthy daughters and am able to perform a job which is both deeply stimulating and rewarding. Only a few years ago many, if not all, of these things would have seemed highly improbable. Without ECT, it is almost certain that they would not have happened.
